# Identification of an adverse outcome pathway (AOP) for chemical-induced craniofacial anomalies using the transgenic zebrafish model

**DOI:** 10.1093/toxsci/kfad078

**Published:** 2023-08-02

**Authors:** Shujie Liu, Toru Kawanishi, Atsuko Shimada, Naohiro Ikeda, Masayuki Yamane, Hiroyuki Takeda, Junichi Tasaki

**Affiliations:** R&D, Safety Science Research, Kao Corporation, Tochigi 321-3497, Japan; Department of Biological Sciences, Graduate School of Science, University of Tokyo, Tokyo 113-0033, Japan; School of Life Science and Technology, Tokyo Institute of Technology, Kanagawa 226-8501, Japan; Department of Biological Sciences, Graduate School of Science, University of Tokyo, Tokyo 113-0033, Japan; R&D, Safety Science Research, Kao Corporation, Kanagawa 210-0821, Japan; R&D, Safety Science Research, Kao Corporation, Tochigi 321-3497, Japan; Department of Biological Sciences, Graduate School of Science, University of Tokyo, Tokyo 113-0033, Japan; Faculty of Life Sciences, Kyoto Sangyo University, Kyoto 603-8555, Japan; R&D, Safety Science Research, Kao Corporation, Kanagawa 210-0821, Japan

**Keywords:** craniofacial anomalies, adverse outcome pathway (AOP), cranial neural crest cells, teratogen, transgenic zebrafish model

## Abstract

Craniofacial anomalies are one of the most frequent birth defects worldwide and are often caused by genetic and environmental factors such as pharmaceuticals and chemical agents. Although identifying adverse outcome pathways (AOPs) is a central issue for evaluating the teratogenicity, the AOP causing craniofacial anomalies has not been identified. Recently, zebrafish has gained interest as an emerging model for predicting teratogenicity because of high throughput, cost-effectiveness and availability of various tools for examining teratogenic mechanisms. Here, we established zebrafish *sox10-EGFP* reporter lines to visualize cranial neural crest cells (CNCCs) and have identified the AOPs for craniofacial anomalies. When we exposed the transgenic embryos to teratogens that were reported to cause craniofacial anomalies in mammals, CNCC migration and subsequent morphogenesis of the first pharyngeal arch were impaired at 24 hours post-fertilization. We also found that cell proliferation and apoptosis of the migratory CNCCs were disturbed, which would be key events of the AOP. From these results, we propose that our *sox10-EGFP* reporter lines serve as a valuable model for detecting craniofacial skeletal abnormalities, from early to late developmental stages. Given that the developmental process of CNCCs around this stage is highly conserved between zebrafish and mammals, our findings can be extrapolated to mammalian craniofacial development and thus help in predicting craniofacial anomalies in human.

Craniofacial anomalies comprise over one‐third of all congenital birth defects and over 700 disorders are associated with craniofacial features (https://www.ncbi.nlm.nih.gov/omim). These anomalies are thought to be caused by genetic and environmental factors, including pharmaceuticals and chemical agents, during embryonic development (Beaty *et al.*, 2016; Edison and Muenke, 2003; Poswillo, 1988; Yoon *et al.*, 2016). Such pharmaceuticals and chemical agents are called teratogens. The teratogenic potential, or teratogenicity, is evaluated and assessed by developmental toxicity tests. Recently the growing number of produced chemicals demands their evaluation and assessment with high-throughput and greater accuracy. Although mammals such as rodents and rabbits have been traditionally used for the tests, current trends toward the 3R principles (replacement, reduction, and refinement) and saving resources have encouraged the development of alternative teratogenicity testing methods (Strähle *et al.*, 2012). To develop an alternative testing strategy for teratogenicity, we definitely need an adverse outcome pathway (AOP) framework which would improve prediction including cross-species extrapolation, and contribute to human health risk assessment. Therefore, a central issue is identification of AOPs that are relays of sequential events with a causal relationship at different levels of biological response leading to a toxic effect (Ankley *et al.*, 2010; Burden *et al.*, 2015; Knapen *et al.*, 2015; Tollefsen *et al.*, 2014; Villeneuve *et al.*, 2014). However, the AOP for chemical-induced craniofacial anomalies has not been fully described.

In the last decade, zebrafish has been considered as a promising model of an alternative method of teratogenicity testing (Bambino and Chu, 2017; He *et al.*, 2014; Nishimura *et al.*, 2016; Teixidó *et al.*, 2019; Yamashita *et al.*, 2014). Zebrafish has several experimental advantages for high-throughput genetic and chemical screening, including evolutionarily conserved developmental programs, various tools for genetic manipulation and rapid external development during the embryonic stage. Accumulating evidence has indicated that the zebrafish model has a high predictive ability for teratogenicity (Augustine-Rauch *et al.*, 2016; Brannen *et al.*, 2010; Inoue *et al.*, 2016; Selderslaghs *et al.*, 2012). Thus, the zebrafish model enables us to describe an AOP of developmental anomalies precisely and develop alternative methods for evaluating teratogenicity based on the AOP.

Recently, by using zebrafish, we demonstrated that craniofacial anomalies caused by teratogens are associated with defects in neural crest cells (NCCs) and their derivatives. Furthermore, these craniofacial anomalies phenocopied neurocristopathy which is a pathology in human caused by the defects in development, migration, or differentiation of NCCs (Liu *et al.*, 2020; Narumi *et al.*, 2020). These results strongly suggest that the AOP of craniofacial teratogenicity is conserved between zebrafish and mammals. Thus, we hypothesized that the developmental process of NCCs would include key events for describing the AOP of teratogenicity. To examine the behavior of NCCs, visualization of these cells with a genetic marker is crucial. *sox10* has been characterized as a common neural crest marker in vertebrates, including mammals and fish (Aoki *et al.*, 2003; Carney *et al.*, 2006; McKeown *et al.*, 2005; Meulemans and Bronner-Fraser, 2004; Simões-Costa and Bronner, 2015; Southard-Smith *et al.*, 1998). Thus, we expected that we can monitor NCC development with transgenic zebrafish lines expressing a fluorescent protein driven by the *sox10* promoter.

Here, we developed a series of transgenic zebrafish lines, *sox10:EGFP*, *sox10:EGFP-CAAX*, and *sox10:Dendra2*, which visualize NCCs. In these lines, the fluorescence demarcated cranial neural crest cells (CNCCs), which migrate from the neural tube along the stereotypical pathway to form the first pharyngeal arch (PA1) and differentiate into craniofacial cartilages such as the ethmoid plate (zebrafish palate) and upper and lower jaws. We administered teratogens known to cause craniofacial anomalies in mammals to the transgenic embryos, and examined the teratogenic effect on CNCC development and craniofacial development. All of the teratogens induced craniofacial anomalies such as cleft palate and micrognathia at 96 hours post-fertilization (hpf), as highlighted by the EGFP fluorescence. We further found that impaired migration of CNCCs and PA1 formation during the first 24 h of development are the early major phenotypes associated these later craniofacial anomalies. Considering that zebrafish embryos around these developmental stages have similar morphological and molecular characteristics compared with mammalian embryos, we postulate that the CNCC migration period is critical for inducing craniofacial anomalies across vertebrates. Based on these results, we propose the AOP of craniofacial anomalies centered by CNCC development. Furthermore, *sox10* transgenic lines are ideal for an AOP-based teratogenicity model for evaluating and predicting craniofacial anomalies.

## Materials and methods

###  

####  

##### Zebrafish maintenance

The zebrafish (*Danio rerio*) strain RIKEN WT (RW), *Tg*(*-5.0sox10:EGFP*), *Tg(-5.0sox10:EGFP-CAAX)*, and *Tg(-5.0sox10:Dendra2)* (RW background) were maintained with a 14-h light/10-h dark cycle. The water temperature was kept at 28°C ± 1°C and water quality conditions were maintained according to the Zebrafish Book (Westerfield, 2000) and the Guide for the Care and Use of Laboratory Animals 8th edition (National Research Council, 2011).

##### Egg production and embryo exposure

Adult male and female zebrafish (4–10 months after fertilization) were placed in a breeding tank with a separator in the late afternoon of the day before spawning. The separator was removed in the morning and spawning was stimulated when the light was turned on. Fertilized eggs were collected within 1 h after removal of the separator. The eggs were incubated in E3 medium (5 mM NaCl, 0.17 mM KCl, 0.33 mM CaCl_2_, 0.33 mM MgSO_4_, 0.1 mM NaOH) at 28°C and exposed to test compounds at 4 hpf. The exposure medium was replaced daily and samples were collected from the 5–6 somite stage (ss) until 96 hpf.

##### Test compounds

The test compounds and exposure concentrations were determined based on Liu *et al.* (2020) and Narumi *et al.* (2020). The compounds and exposure concentrations were as follows: valproic acid (VPA) (15 µM, Wako), salicylic acid (SA) (400 µM, Wako), and caffeine (CAF) (0.5 mM, Wako), which were diluted from stock solutions prepared with distilled water (Life Technologies). Warfarin (WAF) (30 µM, Wako) and methotrexate (MTX) (200 µM, Wako) were diluted from stock solutions prepared with dimethyl sulfoxide (DMSO, Wako).

##### Immunofluorescence staining and fluorescence imaging

Immunofluorescence staining was performed as described (Narumi *et al.*, 2020), with minor modifications. Zebrafish embryos were fixed with 4% paraformaldehyde in phosphate‐buffered saline (PBS) (Wako) at the 10 ss, 20 ss, 24 hpf, and 96 hpf, and then treated with 100% ice-cold methanol (Wako) to achieve dehydration and kept at −20°C for longer storage. The fixed embryos except those being sampled at 96 hpf were permeabilized with 1% Triton X-100 (Cayman Chemical) in PBS (Invitrogen) for more than 1 h before blocking with 3% bovine serum albumin (BSA, Wako) in PBS-T (PBS containing 0.1% Triton X-100), whereas the 96 hpf samples were prepared by following Narumi *et al.* (2020). After the blocking with 3% BSA in PBS-T for 2 h, the samples were incubated with mouse anti-GFP (1/1000, Invitrogen: AB_221568; or 1/1000, EMD Millipore: MAB3580), mouse anti-dendra2 (1/500, Thermo Fisher Scientific: TA180094), mouse anti-collagen type II (anti-coll2, 1/100, DSHB: AB_528165), rabbit anti-active caspase3 (1/1000, BD Pharmingen: 559565), and rabbit anti-phospho-histone H3 (pH3) (Ser10) (1/1000, EMD Millipore: 06-570) primary antibody or PNA lectin conjugated with Alexa Fluor 488 (1/1000, Thermo) overnight at 4°C. The samples were washed 6 times with PBS-T for 15 min, and stained with the following secondary antibodies: Alexa Fluor 488-goat anti-mouse, Alexa Fluor 568-goat antimouse or 568-goat anti-rabbit, or Alexa Fluor 647-goat antimouse secondary antibodies (1/1000, Life Technologies), and DAPI solution (1/1000, DOJINDO) overnight at 4°C. After the samples were then washed 6 times with PBS-T for 15 min, they were embedded in 1% low-melting agarose and mounted on a 35-mm noncoated glass-bottom dish (Matsunami). For live imaging, the samples were anesthetized with 0.02% MS-222 (Sigma-Aldrich) and embedded in 1% low-melting agarose (Sigma-Aldrich) containing 0.02% MS-222 on the glass bottom dish. All immunofluorescence images were acquired using a Zeiss LSM880 or LSM800 system equipped with Zeiss ZEN black or blue software. All procedures were performed at room temperature unless otherwise specified.

For time-lapse imaging, samples were pretreated with VPA (30 µM) for 30 min prior to sample embedding. The samples were anesthetized with 0.02% MS-222 and embedded in 1% low-melting agarose containing 0.02% MS-222 and the teratogens on a 4-chambered glass bottom dish (Greiner). Subsequently, the E3 medium containing MS-222 and teratogens at the same concentrations as above was additionally applied onto the samples.

##### Transgenesis

The Tol2 transposon system was used for the transgenesis (Suster *et al.*, 2009). The promoter region of *sox10* was isolated from RW strain. The promoter region located in the genome 5.0 kb upstream from the translation initiation site was amplified by PCR. The isolated promoter region and EGFP, EGFP-CAAX, or Dendra2 were cloned into pT2AL200R150G by In-Fusion cloning (Takara) to load a transposon cassette according to the manufacturer’s protocol. For Tol2 transposase synthesis, pCS-zT2TP was linearized by Not I restriction enzyme (Takara) and Tol2 transposase mRNA was synthesized using the mMESSAGE mMACHINE SP6 Transcription Kit (Invitrogen). Each transposon construct and Tol2 transposase mRNA were coinjected at the 1-cell embryo stage.

##### Lineage tracing

For lineage tracing of neural crest cells, *Tg(-5.0sox10:Dendra2)* was used. The samples were anesthetized with 0.02% MS-222 and embedded in 1% low-melting agarose containing 0.02% MS-222. To photoconvert the frontonasal region, maxillary region and mandibular region in *Tg(-5.0sox10:Dendra2)* at 10 ss and 24 hpf, each ROI (region of interest) was selected using ZEN blue software on a Zeiss LSM800 confocal microscope and exposed to 10% UV (405 nm) laser for 30 s. Successful photolabeling was confirmed by specific photoconversion of Dendra2 from green to red in each ROI.

##### Quantification of CNCC migration, proliferation, and apoptosis

To quantitatively assess the migration of CNCCs, we measured the linear distance between the midbrain-hindbrain boundary (MHB) and the anterior border of the CNCC population migrating via the frontonasal pathway at 10 ss (for details, see Figure 3A) using Fiji measurement plugin (National Institutes of Health). To quantify mitotic CNCCs, we counted the number of the cells that displayed positive signals for both pH3 and EGFP anterior to the MHB at 10 ss (magenta region in Figure 6A) or within the PA1 at 24 hpf. Similarly, for quantifying apoptotic CNCCs, we counted the number of the cells that displayed double-positive signals for both active-Caspase 3 and EGFP in the same regions as above. In both experiments, we used z-stack images obtained by confocal microscopy and counted the number of mitotic and apoptotic cells distributed on the left side of the embryos.

##### Statistics

Multiple comparison tests were performed using Graph Pad Prism version 8 software for Windows (La Jolla). *p*-values were calculated using a 1-way ANOVA followed by Dunnett’s multiple comparison tests for quantification of the migration distance of the pH 3- or Active Cas3-positive CNCCs. *p*-values < .05 were considered statistically significant. All data are presented as the mean ± SD unless otherwise specified.

## Results

###  

#### sox10 reporter lines visualize the developmental process of neural crest cells

**Figure 1.**
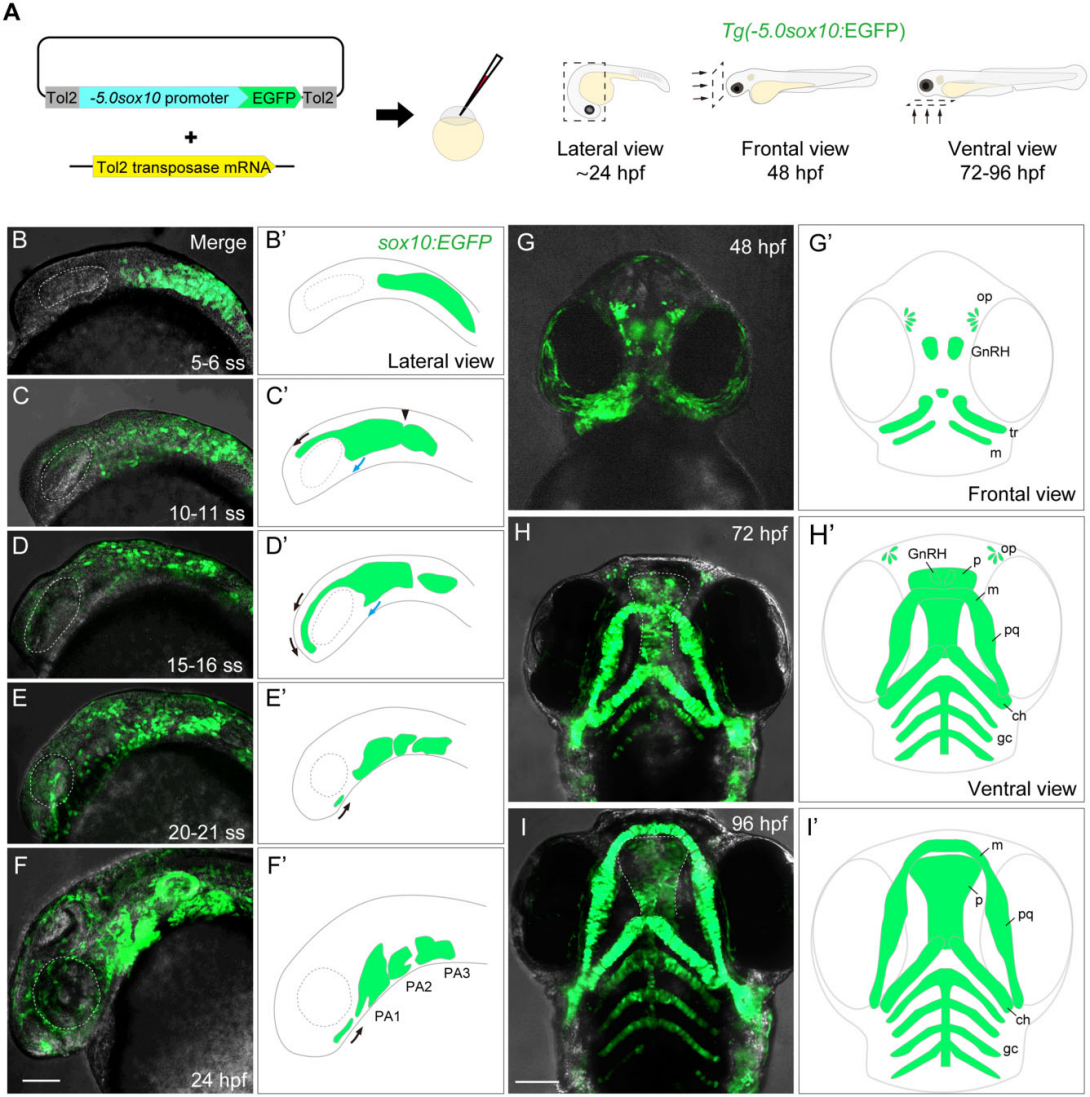
Generation and characterization of the *sox10:EGFP* transgenic zebrafish. (A) Schematic diagram for generating *Tg(-5.0sox10:EGFP)* (left) and directions of view for imaging embryos shown in (B)–(I) (right). (B–I′) Live imaging of *sox10:EGFP* embryos (B–I) and schematic diagram of the EGFP distribution (B′–I′). (B, B′) EGFP expression was detected at the 5–6 ss in premigratory and migratory neural crest cells. (C–E′) EGFP expression was detected in CNCCs which migrated from the anterior-most midbrain and the hindbrain to the pharyngeal pouch via frontonasal (black arrows) and maxillary (blue arrows) pathways at 10–11 ss (C, C′), 15–16 ss (D, D′) and 20–21 ss (E, E′). The arrowhead in C′ shows the midbrain-hindbrain boundary (MHB). (F, F′) EGFP-positive CNCCs formed pharyngeal arches (PAs) at 24 hpf. (G, G′) EGFP-positive CNCCs formed the primordium of craniofacial skeletal elements (the palate and lower jaw) and placodes (olfactory neurons and GnRH neurons) at 48 hpf. (H, H′) EGFP-positive CNCCs were observed in the craniofacial skeletal elements and sensory neurons at 72 hpf. (I, I′) EGFP-positive skeletal elements grew and eventually composed the face at 96 hpf. ch, ceratohyal; gc, gill cartilages; GnRH, gonadotropin-releasing hormone neuron; m, Meckel’s cartilage; op, olfactory placode; p, palate; PA, pharyngeal arch; pq, palatoquadrate; tr, trabeculae. Scale bar: 100 µm.

To visualize the developmental process of NCCs in zebrafish, we generated *Tg(-5.0sox10:EGFP)*, *Tg(-5.0sox10:EGFP-CAAX)*, and *Tg(-5.0sox10:Dendra2)* (referred to as *sox10:EGFP*, *sox10:EGFP-CAAX*, and *sox10:Dendra2*, respectively) lines using the Tol2 transposon system (Figure 1A and Table 1). EGFP expression was detected as early as the 5–6 ss in premigratory and migratory NCCs by live imaging (Figs. 1B and 1B′). At the 10–21 ss, the fluorescence highlighted cranial NCCs (CNCCs), which migrated from the anterior midbrain and hindbrain to the pharyngeal pouches via the frontonasal pathway (black arrows) and maxillary pathway (blue arrows), respectively (Figs. 1C–E′). The migratory CNCCs via the frontonasal pathway reached the ventral side of the eye at around the 21 ss (Figure 1E′ black arrow). The migratory CNCCs expressing EGFP reached the ventral side of the pouches and began to form the pharyngeal arches (PAs) at 24 hpf (Figs. 1F and 1F′). The PA1, which give rise to a large part of the craniofacial bones, were EGFP-positive at this stage (Figure 1F′). The migratory CNCCs via the frontonasal pathway moved toward the PA1 (Figure 1F′ black arrow). *sox10:EGFP* continuously visualized the developmental process of CNCCs and craniofacial morphogenesis from 48 to 96 hpf (Figs. 1G–I′). The EGFP-expressing CNCCs were present in the developing craniofacial skeletal primordium; trabeculae (developing lateral part of the ethmoid plate), median element (developing medial part of the ethmoid plate) derived from frontonasal process, and Meckel’s cartilage (lower jaw) at 48 hpf (Figs. 1G and 1G′). At this stage, the EGFP-expressing CNCCs were also present in olfactory placodes and gonadotropin-releasing hormone cells (Figs. 1G and 1G′). The skeletal primordium differentiated into the ethmoid plate (zebrafish palate) consisting of the trabeculae and median elements and Meckel’s cartilage at 72 hpf (Figs. 1H and 1H′). These structures grew and each skeletal element was firmly formed at 96 hpf (Figs. 1I and 1I′).

**Table 1.** Established transgenic lines

Transgenic line	Fluorescent protein localization	Purpose
*sox10:EGFP*	Cytoplasm	Cell morphology
*sox10:EGFP-CAAX*	Endomenbrane	Cell morphology
*sox10:Dendra2*	Cytoplasm	Lineage tracing

Three transgenic lines were established. Each line showed distinct fluorescence localization and utility for a specific purpose.

Based on live-imaging observations, the developmental processes of CNCCs can be roughly classified into 3 periods: the migration period (Figs. 1B–E), PA1 formation period (Figure 1F), and outgrowth period (Figs. 1G–I). Collectively, these results show that our *sox10:EGFP* line demarcates all the CNCC populations reported so far (Carney *et al.*, 2006) and thus can visualize the entire processes of CNCC development throughout embryogenesis. Other transgenic lines, *sox10:EGFP-CAAX* and *sox10:Dendra2*, labeled the same population of cells as *sox10:EGFP* during development (Table 1 and Supplementary Figure 1).

**Figure 2.**
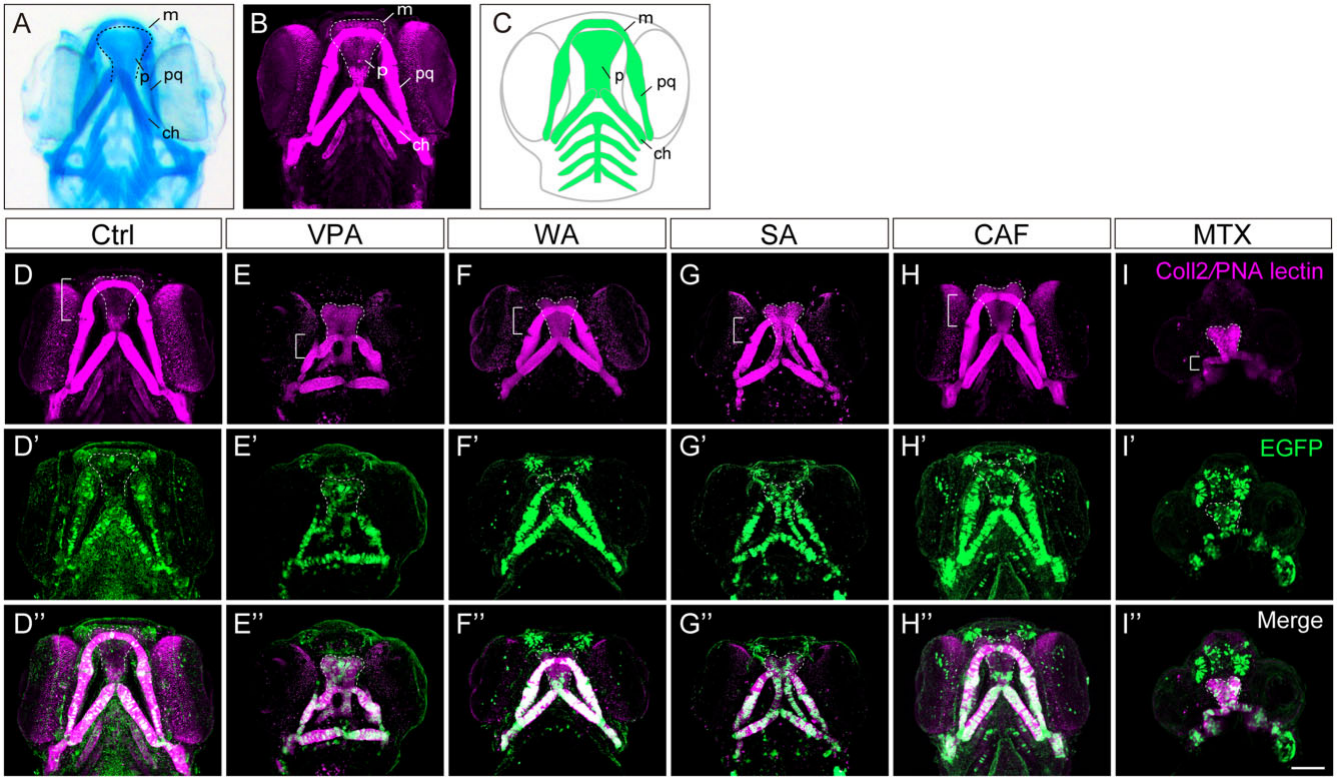
Craniofacial anomalies were identified in teratogen-treated *sox10:EGFP* zebrafish embryos at 96 hpf. (A, B) Craniofacial cartilage stained with Alcian blue (A) and anti-coll2 antibody and PNA lectin (B). (C) Schematic illustration of craniofacial cartilage elements. m, Meckel’s cartilage; p, palate; pq, palatoquadrate; ch, ceratohyal. (D–I″) *sox10:EGFP* embryos were treated with the following teratogens and displayed craniofacial anomalies. (D–D″) Ctrl, E3 control. (E–E″) VPA, valproic acid (15 mM). (F–F″) WAF, warfarin (30 mM). (G–G″) SA, salicylic acid (300 mM). (H–H″) CAF, caffeine (500 mM). (I–I″) MTX, methotrexate (200 mM). Immunohistochemical staining of craniofacial cartilage was performed with anti-coll2 antibody and PNA lectin (D–I), and anti-GFP antibody (D′–I′) following the teratogen exposure. Anti-GFP staining was co-merged with the cartilage staining (D″–I″). Control: *n* = 25, VPA: *n* = 18, WAF: *n* = 23, SA: *n* = 16, CAF: *n* = 31, MTX: *n* = 26. Bracket indicates the lower jaw of zebrafish. Dashed lines indicate outline of the palate. All images were taken from ventral view. m, Meckel’s cartilage; p, palate; pq, palatoquadrate. Scale bar: 100 µm.

Next, we examined whether these lines are useful for detecting craniofacial anomalies (Figure 2). For this, we tested them with 5 teratogens (VPA, WAF, SA, CAF, and MTX). These teratogens are known to cause craniofacial defects in mammals and zebrafish (Liu *et al.*, 2020; Narumi *et al.*, 2020). To visualize cartilage, we immunostained treated embryos using both an anti-coll2 antibody and PNA lectin, which recapitulates Alcian blue staining (Figs. 2A–C), and compared the fluorescence pattern with EGFP expression pattern. All 5 teratogens induced craniofacial defects in cartilage at 96 hpf, such as cleft palate and small jaws (micrognathia), as reported in our previous studies (Figs. 2D–I; Liu *et al.*, 2020; Narumi *et al.*, 2020). The craniofacial anomalies showed similar patterns among the teratogens; however, the phenotypic severity was dependent on the teratogen. Importantly, the EGFP patterns mostly overlapped with the cartilage staining in treated embryos, and thus these defects were also detected by EGFP expression (Figs. 2D′–I″).

Taken together, our results show that *sox10:EGFP* can visualize the anomalies of the craniofacial structure as precisely as the conventional cartilage staining. The same craniofacial defects were observed in *sox10:EGFP-CAAX* and *sox10:Dendra2* embryos (Supplementary Figs. 2 and 3). Remarkably, the transgenic lines have a great advantage that every key event of CNCC development in an individual embryo can be traced by live imaging, starting from the emergence of CNCCs, which is essential to identify AOPs. Using these transgenic lines, we will describe possible key events for the CNCC-related AOP.

#### Inhibitory effect of the teratogens on neural crest cell migration

**Figure 3.**
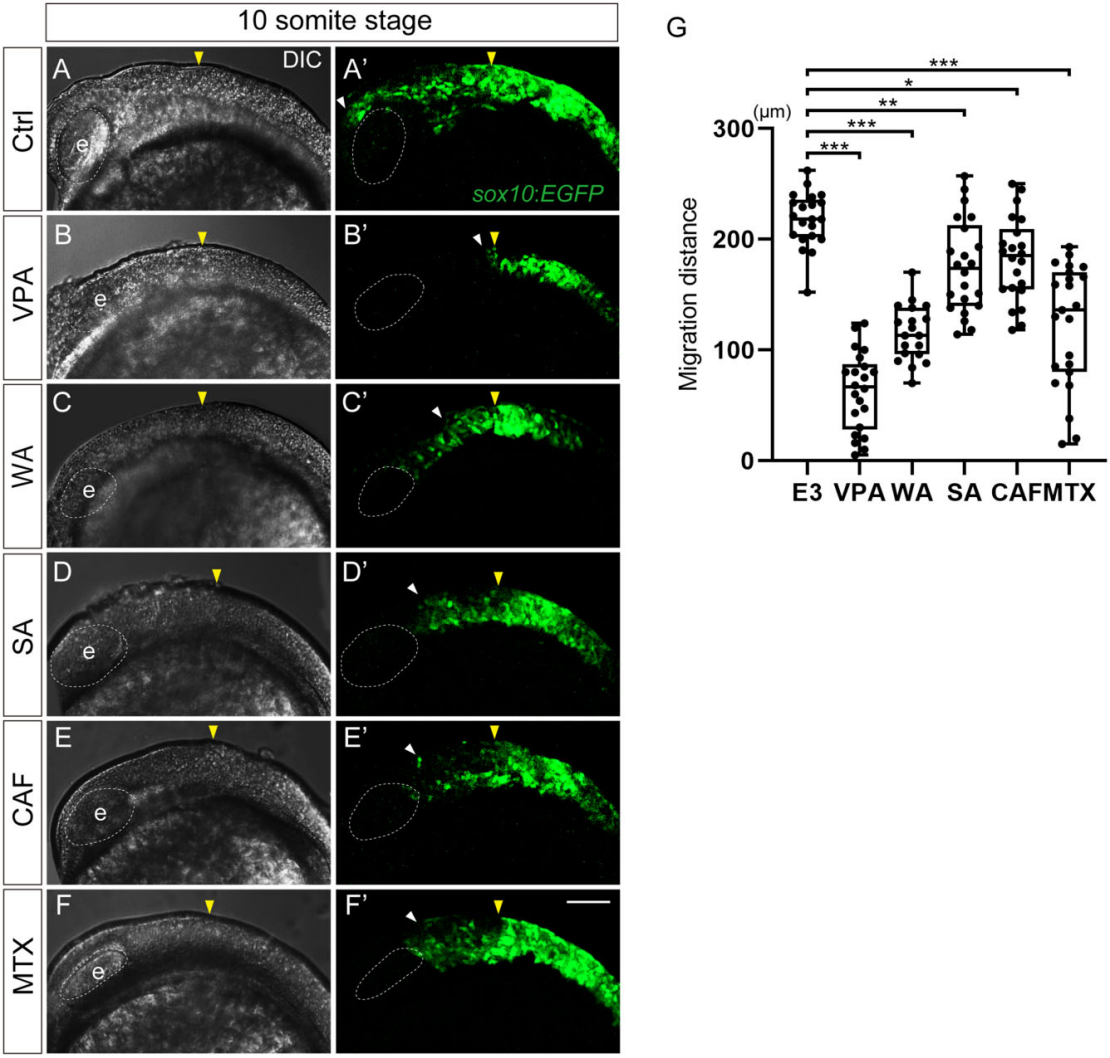
CNCC migration was inhibited in teratogen-treated *sox10:EGFP* embryos. (A–F′) Migratory CNCCs were visualized in *sox10:EGFP* embryos at 10 ss. The yellow arrowheads show the position of the midbrain-hindbrain boundary(MBH). The white arrowheads represent the anterior border of the migratory CNCCs. All teratogens inhibited CNCC migration. (G) Quantification of the distance between the MHB (yellow arrowheads) and the anterior border of the migratory CNCCs via the frontonasal pathway (white arrowheads). Control: *n* = 21, VPA: *n* = 22, WA: *n* = 19, SA: *n* = 22, CAF: *n* = 22, MTX: *n* = 23. *p < .05, **p < .01, ***p < .001 (1-way ANOVA followed by Dunnett’s multiple comparison test). e: eye. Scale bar: 100 μm.

Our previous study suggested that craniofacial defects induced by teratogens are consequences of their adverse effects on CNCC development (Liu *et al.*, 2020; Narumi *et al.*, 2020). To elucidate the key events that trigger craniofacial malformation, we focused on the earliest phase of CNCC development, when the cartilages are yet to be formed. We focused on the migration period and analyzed the effects of the teratogens on migratory CNCCs by live imaging of *sox10:EGFP* embryos at 10 ss. In control embryos, CNCCs migrated from the midbrain and anterior hindbrain region at 10 ss toward the dorsal side to the eye along the frontonasal pathway or toward the pharyngeal region along the maxillary pathway. However, CNCC migration via both pathways was inhibited by all 5 teratogens tested (Figs. 3B–F′). To quantify inhibition of cell migration, we focused on the CNCCs migrating via the frontonasal pathway and measured the distance between the MHB (yellow arrowheads in Figure 3) and the anterior border of the migratory CNCCs (white arrowheads in Figure 3). The migration distance of migratory CNCCs was significantly reduced in the teratogen-treated groups (Figure 3G). Furthermore, all teratogens exhibited dose dependency in their inhibitory effects on CNCC migration (Supplementary Figs. 4A–E). Indeed, time-lapse imaging of zebrafish embryos treated with a higher dose of VPA, as an example, revealed a pronounced inhibition of CNCC migration (Supplementary Movies 1 and 2). Interestingly, there was a correlation between the extent of the inhibitory effect on migration and the morphological severity of craniofacial anomalies, ie, cleft palate and micrognathia, at 96 hpf (Figs. 2D–I″). Regarding MTX treatment, however, the migration phenotype was milder compared with the morphological phenotype, especially for micrognathia, at 96 hpf (Figs. 2I–I″) (Liu *et al.*, 2020).

We have previously reported that the expression level of *sox10* was disturbed by teratogen exposure, as determined by RT-PCR analysis (Liu et al., 2020). Consistently, as shown in Supplementary Figure 4, the EGFP signal tends to decrease as the concentration of teratogens increase. In spite of this, the intensity of the fluorescence signals at high-dose concentrations was sufficient enough for us to investigate CNCC behavior.

These results suggest that a defect in CNCC migration is the earliest key event caused by the teratogens and that this inhibitory effect on CNCC migration account for disruption of or abnormal CNCC development in the later events.

#### Developmental fate of the CNCCs in the PA1 and frontonasal prominence

**Figure 4.**
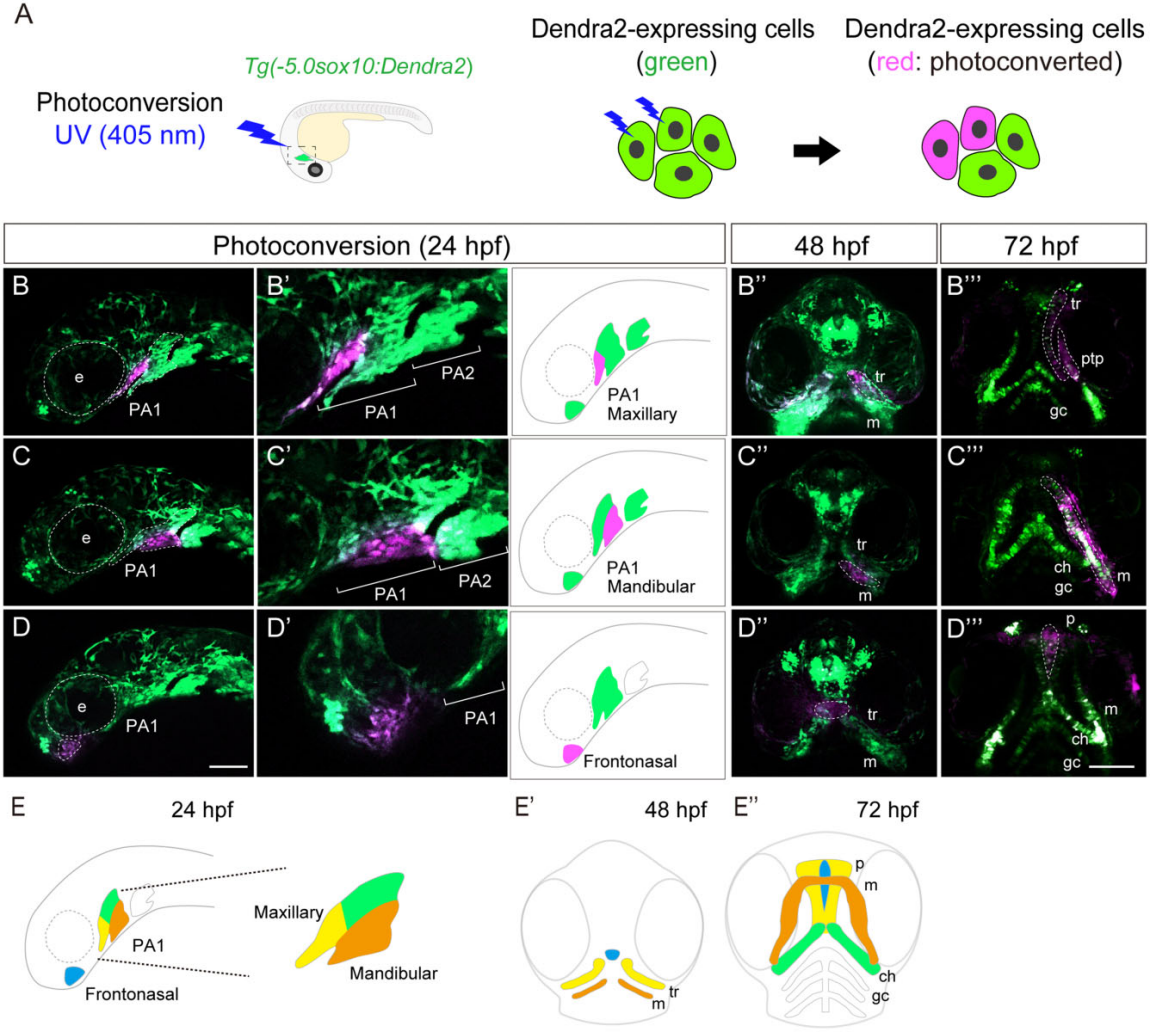
Lineage tracing of CNCCs in PA1 and frontonasal prominence using *sox10:Dendra2*. (A) Photoconversion was performed in *sox10:Dendra2* transgenic zebrafish. Photoconvertible fluorescent protein Dendra2 is photoactivated by UV light (405 nm) from green to red. Specific Dendra2-expressing cells were labeled irreversibly and traced. (B–D′″) Lineage tracing of the PA1 components: maxillary prominence (B, B′), mandibular prominence (C, C′), and frontonasal prominence (D, D′). Magnified view of the photoconverted each prominence was displayed in panel B′, C′, and D′. (B–B′″) The maxillary prominence was labeled at 24 hpf and differentiated into lateral part of the ethmoid plate (zebrafish palate) and the pterygoid process (ptp), which is the upper jaw at 72 hpf. (C–C′″) The mandibular prominence was labeled at 24 hpf and differentiated into Meckel’s cartilage of the lower jaw. (D–D′″) The frontonasal prominence was labeled at 24 hpf and differentiated into the medial part of the palate at 72 hpf. (E–E″) The schematic diagram of the lineage tracing of the PA1 and frontonasal prominence. Each color in the panel E represents the following: yellow indicates the photoconverted region of the maxillary prominence, orange indicates the photoconverted region of the mandibular prominence, and blue indicates the photoconverted region of the frontonasal prominence. ch, ceratohyal; e, eye; gc, gill cartilages; m, Meckel’s cartilage; mx, maxillary; p, palate; ptp, pterygoid process; tr, trabeculae. Scale bar: 100 µm.

Most of the above affected migratory CNCCs are known to colonize the PA1 under normal conditions. To relate the migratory defects with late craniofacial anomalies, we examined the fate of CNCCs in the PA1 NCCs at later stages by lineage tracing. For this, we utilized a *sox10:Dendra2* transgenic line (Figure 4A and Supplementary Figs. 1C–D′). The photoconvertible fluorescent protein Dendra2 irreversibly changes color from green to red upon photoactivation by UV light (405 nm), and thus a specific CNCC population can be labeled and traced chronologically (Figure 4A).

We first labeled the maxillary prominence of the PA1 at 24 hpf and analyzed their descendants at 48 and 72 hpf (Figs. 4B–B′″). The maxillary prominence eventually became the lateral part of the palate (trabeculae) and pterygoid process (upper jaw) (Figure 4B′″). Next, we performed the same analysis for the mandibular prominence of the PA1 (Figs. 4C–C′″). The mandibular prominence differentiated into the Meckel’s cartilage (lower jaw) (Figure 4C′″). Furthermore, when we labeled the frontonasal prominence at 24 hpf (Figure 4D), the labeled cells gradually occupied the medial part of the palate at 48 and 72 hpf (Figs. 4D″ and 4D′″). Thus, the transgenic lines were able to identify the morphological changes that lead to the craniofacial anomalies (Figs. 4E–E″), and revealed the lineage-relationship between CNCCs in the PA1 and later craniofacial skeletons.

#### Morphological changes in the PA1 are correlated with craniofacial anomalies in sox10:EGFP embryos

**Figure 5.**
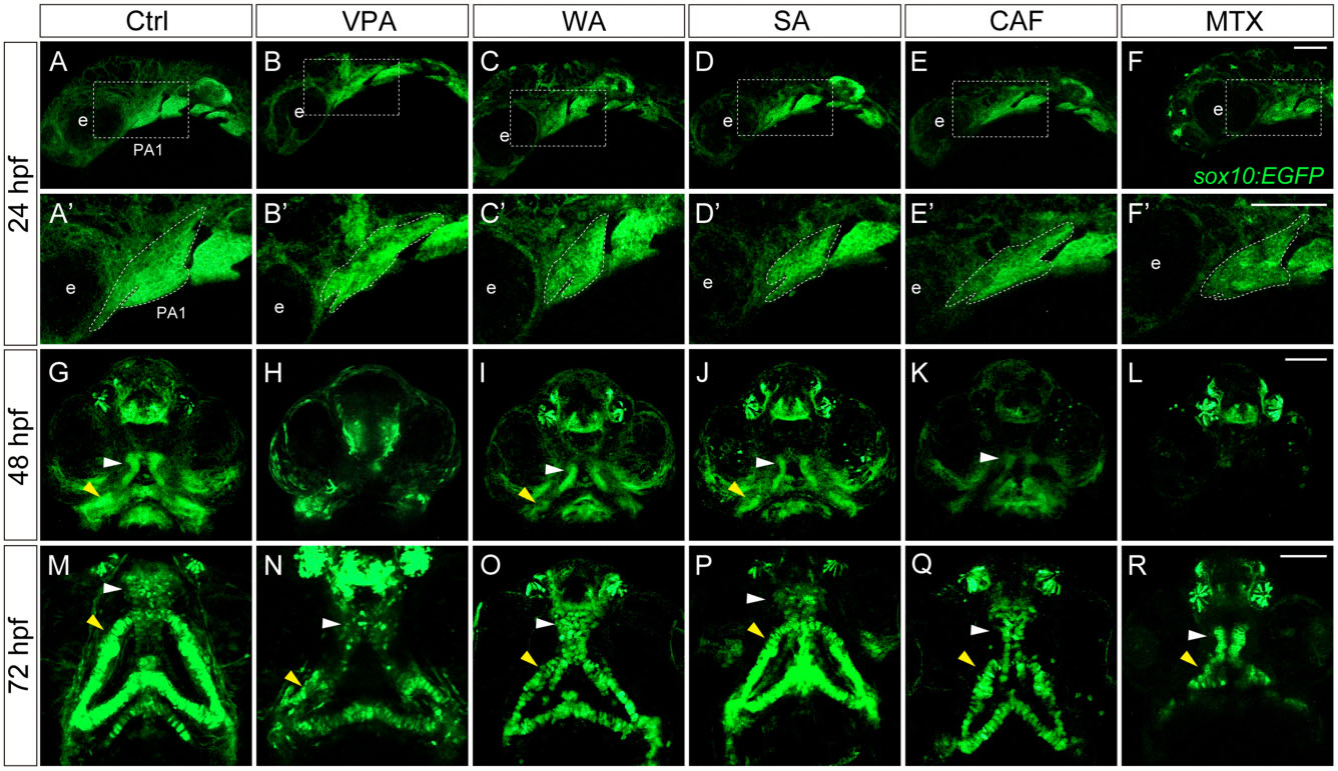
Changes in the morphology of craniofacial anomalies in teratogen-treated *sox10:EGFP* embryos. (A–F) Lateral live view of the first pharyngal arch (PA1) at 24 hpf. (A′–F′) Enlarged view of the white rectangle in panel A–F. Abnormal morphology of the PA1 was observed in the teratogen-treated embryos. (G–L) The developing palates (white arrowheads) and mandibles (yellow arrowheads) were disrupted in the teratogen-treated embryos at 48 hpf. (M–R) The PA1 defects resulted in a small-sized palate with clefting (cleft palate) and shortening in the Meckel’s cartilage at 72 hpf. e: eye. Scale bar: 100 µm.

To obtain the direct relationship between PA1 and teratogen-induced craniofacial phenotypes, we examined how craniofacial anomalies emerge by focusing on the PA1 formation period and outgrowth period. We treated *sox10:EGFP* embryos with the teratogens and performed live-imaging analysis of the PA1 morphology at 24 hpf, craniofacial placode formation at 48 hpf and craniofacial morphology at 72 hpf (Figs. 5A–R). Teratogen-exposed *sox10:EGFP* embryos displayed PA1 defects such as truncated maxillary and mandibular prominences at 24 hpf (Figs. 5A–F′). At 48 hpf, the primitive palate (white arrowhead) and lower jaw (yellow arrowhead) were disrupted by the teratogens, leading to hypoplasia or aplasia of the structures (Figs. 5G–L). At 72 hpf, the treated embryos exhibited the palate hypoplasia with a cleft (cleft palate) and shortening in Meckel’s cartilage (micrognathia) (Figs. 5M–R). WA-, SA-, and CAF-treated embryos showed mild phenotypes, namely, hypoplasia of the palate and mandible, whereas VPA- and MTX-treated embryos showed more severe phenotypes, namely, aplasia of the palate and mandible. These results are in agreement with the severity of craniofacial defects in each teratogen (Figs. 2 and 3).

Taken together, our data show that the teratogenic effects observed at 96 hpf originated from aberrant morphological changes observed during the migration (10–21 ss.) and PA1 formation (24 hpf) periods.

#### Altered cell proliferation and apoptosis in the CNCCs during the migration period

What cellular defects underlined teratogen-induced craniofacial anomalies? Proper regulation of cell proliferation and death is crucial in neural crest development during vertebrate embryogenesis (Kee and Bronner-Fraser, 2005; Kulesa *et al.*, 2004). Thus, we hypothesized that teratogen-induced disruption of cell proliferation and apoptosis partially account for the observed migration defects of CNCCs during the migration period. These defects could be key events for the PA1 dysplasia, finally leading to craniofacial anomalies as an adverse outcome. We thus examined cell proliferation and apoptosis in premigratory and migratory CNCCs during the first 24 h of development.

**Figure 6.**
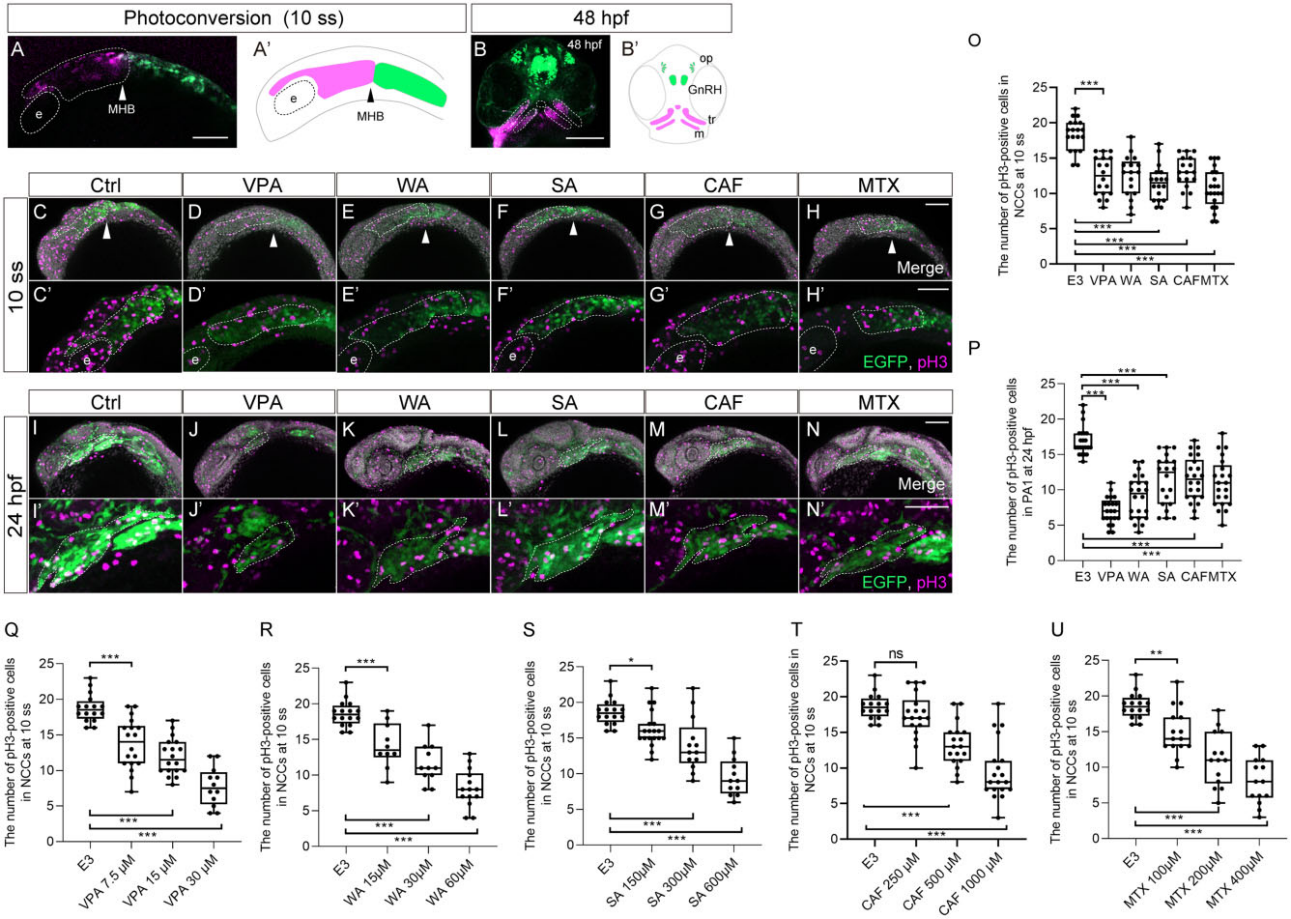
Cell proliferation was decreased in the migratory and premigratory CNCCs and CNCCs in the PA1 by teratogen treatment. (A, B) The anterior CNCCs (magenta in panel A) were labeled at 10 ss and subsequently differentiated into maxillary (trabeculae), mandibular (Meckel’s cartilage) and frontonasal prominence at 48 hpf (magenta in panel B). (A′, B′) A schematic diagram of the lineage tracing of the anterior CNCCs. Magenta and green indicate the photoconverted region and intact region, respectively. White and black arrowheads in panels A and A′ represent the position of the midbrain-hindbrain boundary (MHB). (C–N′) Immunofluorescence images of mitotic premigratory and migratory CNCCs at 10 ss (C–H′) and 24 hpf (I–N′). VPA-, WA-, SA-, CAF-, and MTX-treated embryos were stained with anti-GFP and anti-phospho-histone H3 (pH3) antibodies. Mitotic premigratory and migratory CNCCs were decreased by teratogen treatment (C–H′). (C′–H′) Magnified view of the panels C–H. Mitotic CNCCs in the PA1 were decreased by teratogen treatment. (I′–N′) Magnified view of the panels I–N. Green represents the CNCCs at 10 ss and the CNCCs in the PA1 at 24 hpf. Magenta indicates the mitotic cells stained with anti-pH3 antibody. White dotted lines trace the eye, the region of the anterior CNCCs at 10 ss and PA1 at 24 hpf. (O, P) Quantification of the number of pH3-positive CNCCs in the area at 10 ss (O) and PA1 at 24 hpf (P). *n* = 19 (control), 18 (VPA), 17 (WA), 18 (SA), 17 (CAF), 21 (MTX) in (O). *n* = 24 (control), 22 (VPA), 23 (WA), 24 (SA), 23 (CAF), 20 (MTX) in (P). (Q–U) Dose-dependent decrease of the mitotic activity. Control: *n* = 16, VPA (7.5 µM): *n* = 18, VPA (15 µM): *n* =18, VPA (30 µM): *n* = 12, WA (15 µM): *n* = 10, WA (30 µM): *n* = 11, WA (60 µM): *n* = 14, SA (150 µM): *n* = 19, SA (300 µM): *n* = 13, SA (600 µM): *n* = 12, CAF (250 µM): *n* = 18, CAF (500 µM): *n* = 18, CAF (1000 µM): *n* = 19, MTX (100 µM): *n* = 15, MTX (200 µM): *n* = 14, MTX (400 µM): *n* = 14. ***p < .001 (1-way ANOVA followed by Dunnett’s multiple comparison test). e; eye, GnRH, gonadotropin-releasing hormone neuron; m, Meckel’s cartilage; op, olfactory placode, tr; trabeculae. Scale bar: 100 μm.

To precisely locate premigratory and migratory CNCCs at 10 ss which are to populate the PA1, we again performed the lineage tracing experiments using *sox10:Dendra2* embryos. Because the CNCCs positioned anterior to the MHB migrated to the craniofacial regions by 24 hpf (Figure 3), we reasoned that the CNCCs anterior to the MHB constitute the PA1 derivatives in later stages. To test this, we photoconverted CNCCs in the region of embryos at 10 ss and analyzed the distribution of labeled cells at 48 hpf (Figs. 6A–B′). The photoconverted CNCCs differentiated into the primordium of the palate (trabeculae) and lower jaw (Meckel’s cartilage) as well as a cell population derived from the frontonasal prominence at 48 hpf (Figs. 6B and 6B′), indicating that these premigratory and migratory CNCCs at 10 ss are destined to constitute the PA1.

The above CNCC populations at 10 ss and in the PA1 at 24 hpf were subjected to cell proliferation assay, ie, immunofluorescence staining against pH3 as a mitotic marker (Figs. 6C–N′). Quantification of the number of the pH3-positive premigratory and migratory CNCCs revealed that the mitotic activity was significantly decreased by treatment with any of the teratogens at 10 ss (Figure 6O). Moreover, the mitotic activity at 10 ss exhibited dose-dependent decrease following treatment with all the teratogens (Figs. 6Q–U). These defects in migration behavior and mitotic activity account for the subsequent key event, PA1 formation (Figure 6P).

**Figure 7.**
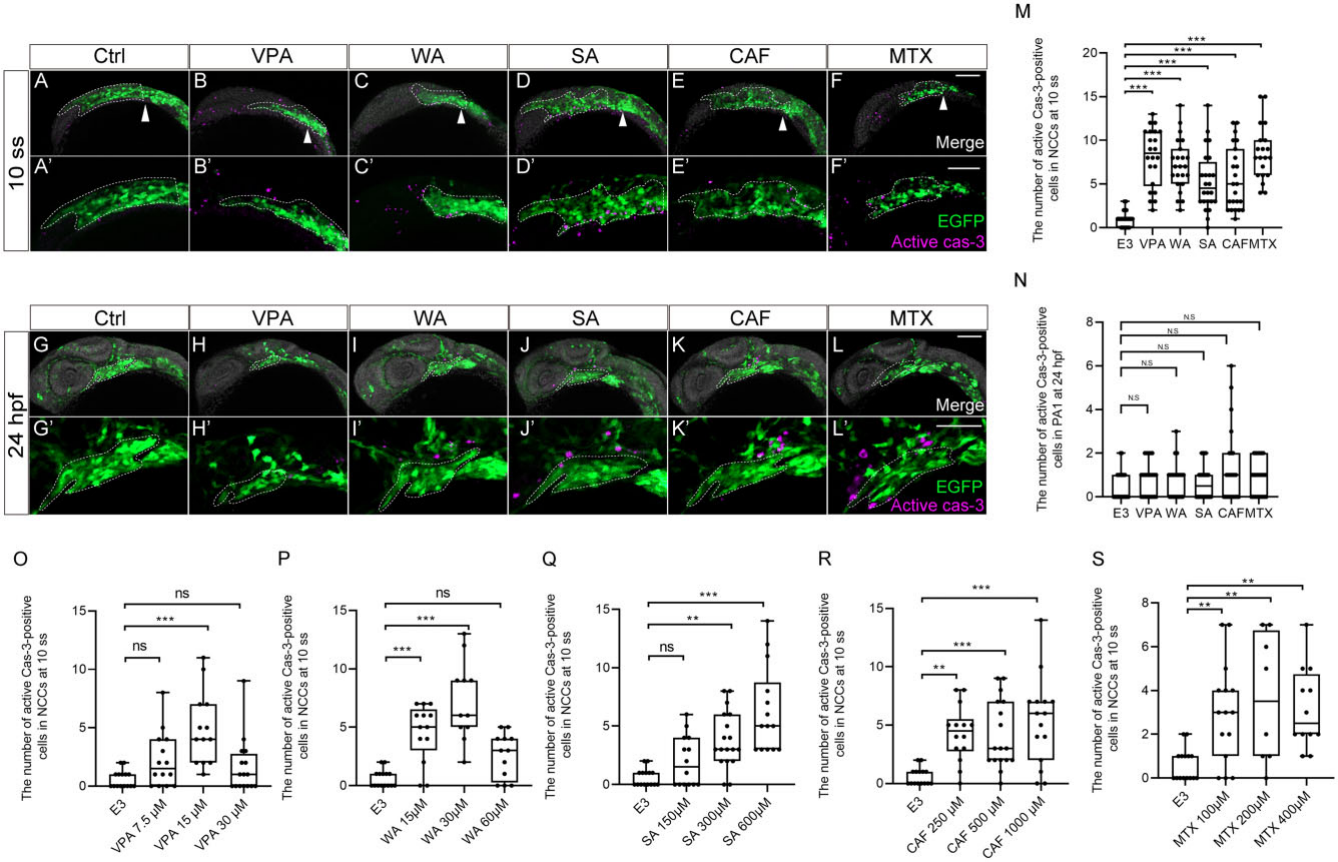
Apoptosis was increased in premigratory and migratory CNCCs, but not in CNCCs in PA1. (A–L′) Immunofluorescence images of VPA, WA, SA, CAF, and MTX-treated embryos that were stained with anti-active caspase 3 (active cas-3) and anti-GFP antibody at 10 ss (A–F′) and 24 hpf (G–L′). (A–F′) Apoptosis in premigratory and migratory CNCCs was increased by teratogen treatment. White arrowheads indicate the midbrain-hindbrain boundary (MHB). (A′–F′) Magnified view of the panels A–F. (G–L) Apoptotic CNCCs in PA1 were not significantly increased by teratogen treatment. (G′–L′) Magnified view of the panels G–L. Green represents the premigratory and migratory CNCCs at 10 ss and the CNCCs in the PA1 at 24 hpf. Magenta indicates the apoptotic CNCCs stained with anti-active cas-3 antibody. White dotted lines trace the eye and the region of the anterior CNCCs at 10 ss and PA1 at 24 hpf. (M, N) Quantitation of the number of active cas-3-positive CNCCs at 10 ss (M) and PA1 at 24 hpf (N). *n* = 22 (control), 21 (VPA), 22 (WA), 20 (SA), 22 (CAF), 21 (MTX) in (M). *n* = 25 (control), 23 (VPA), 20 (WA), 23 (SA), 22 (CAF), 23 (MTX) in (N). (O–S) Dose-dependent increase of apoptotic cells. Control: *n* = 15, VPA (7.5 µM): *n* = 14, VPA (15 µM): *n* =13, VPA (30 µM): *n* = 16, WA (15 µM): *n* = 13, WA (30 µM): *n* = 11, WA (60 µM): *n* = 12, SA (150 µM): *n* = 14, SA (300 µM): *n* = 18, SA (600 µM): *n* = 14, CAF (250 µM): *n* = 14, CAF (500 µM): *n* = 17, CAF (1000 µM): *n* = 15, MTX (100 µM): *n* = 15, MTX (200 µM): *n* = 8, MTX (400 µM): *n* = 12. ***p < .001 (1-way ANOVA followed by Dunnett’s multiple comparison test). e: eye. Scale bar: 100 µm.

Next, we examined apoptosis by active Caspase-3 (Cas-3) antibody staining (Figs. 7A–L′). Quantification of the number of active Cas-3-positive CNCCs indicated that apoptotic cells significantly increased at 10 ss (Figure 7M). Moreover, the number of apoptotic cells exhibited a dose-dependent increase following treatment of all teratogens at 10 ss (Figs. 7O–S). However, at the highest dose of VPA and WA, embryos did not show a significant increase in apoptotic cells (Figs. 7O and 7P). One reason for this result could be that excessive cell death was induced at earlier stages by the high-dosage teratogen treatment. In contrast, apoptosis of CNCCs in the PA1 at 24 hpf was not significantly induced by the teratogen exposure (Figure 7N). Thus, the number of apoptotic CNCCs in premigratory and migratory CNCCs was in agreement with the phenotypic severity at later stages.

Collectively, the data indicated that decreased mitotic ability and increased apoptosis in CNCCs during the migration period could be major key events for teratogen-indued craniofacial anomalies via PA1 dysplasia.

## Discussion

In the present study, we established transgenic lines driven by the *sox10* promoter to visualize CNCCs and examined their cellular behaviors in detail to identify an AOP for chemical-induced craniofacial anomalies. Teratogens affected CNCC migration, mitotic ability, and apoptosis, leading to the PA1 morphological defect. These defects finally resulted in craniofacial anomalies such as cleft palate and micrognathia as adverse outcomes. Our lineage tracing experiments showed that, like in mammals, CNCCs in the zebrafish PA1 differentiated into craniofacial skeletal elements. Thus, the AOP of craniofacial anomalies could be conserved between fish and mammal.

**Figure 8.**
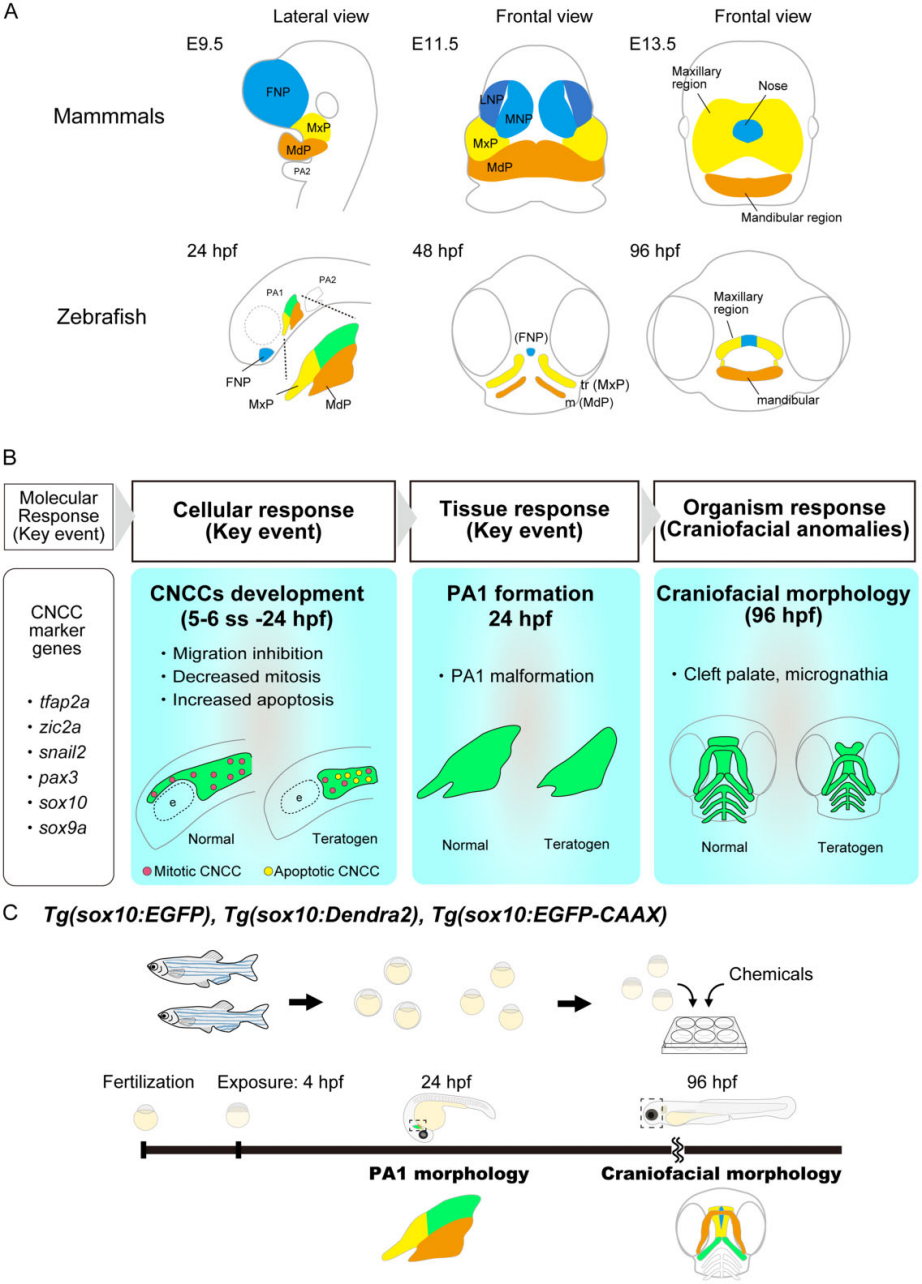
Conserved craniofacial morphogenesis during the pharyngeal stage and the AOP of craniofacial anomalies identified in the present study. (A) Schematic comparison of craniofacial morphogenesis between mammals (mouse) and zebrafish. Zebrafish and mammals share conserved craniofacial morphogenesis. At the pharyngeal stage, the frontonasal prominence (FNP), maxillary prominence (MxP) and mandibular prominence (MdP) are formed in both species. FNP and each region in the PA1 differentiate into craniofacial elements. (B) Identified AOP of chemical-induced craniofacial anomalies. (C) AOP-based teratogenicity assay utilizing the *sox10* transgenic lines. PA1 morphology at 24 hpf and craniofacial morphology at 96 hpf are practical endpoints of teratogenicity evaluation and prediction.

The craniofacial morphology of mammals is apparently different from that of zebrafish at the late embryonic (fetal) stages; however, the pharyngeal period (24 hpf for zebrafish and E9.5 for mouse), when PAs are being formed, is conserved at the morphological and transcriptome level among vertebrates (Irie and Kuratani, 2011; Kuratani, 2005). During this period, one of the crucial events is the development of NCCs (Etchevers *et al.*, 2019; Hockman *et al.*, 2019; Simões-Costa and Bronner, 2013). CNCCs originate from the midbrain and anterior hindbrain region during neurulation (Chai *et al*., 2000; Rocha *et al.*, 2020; Figs. 1B and 1B′). CNCCs migrate stereotypically along the frontonasal or maxillary pathways and form the frontonasal prominence and the PA1 (the maxillary and mandibular prominence), respectively (Chai *et al.*, 2000; Rocha *et al.*, 2020; Serbedzija *et al.*, 1990; Figs. 4E–4E″ and 8A). The frontonasal prominence forms the facial midline, whereas the PA1 forms the mandibular region (lower jaw including Meckel’s cartilage) and the maxillary region (palate) in both mouse and zebrafish (Figure 8A) (Clouthier *et al.*, 2010; Graham, 2001; Graham and Richardson, 2012; Knight and Schilling, 2006). Thus, key events constituting AOPs of craniofacial anomalies should be conserved during this period between fish and mammals.

Similarities between fish and mammalian craniofacial development are also observed even after the pharyngeal period. In mammals, one of the critical events causing craniofacial anomalies is a failure of the primary and secondary palate formation (palatogenesis) during late craniofacial morphogenesis (E11.5 for mouse embryos). The primary palate develops from the frontonasal prominence and is transiently formed anterior to the secondary plate (Figure 8A). The secondary palate develops from the maxillary prominence and is finally fused with the primary palate to complete the process of craniofacial morphogenesis (Bush and Jiang, 2012). Any disturbance of this process in mammals can cause orofacial clefts (Hammond and Dixon, 2022; Lan *et al.*, 2015). In zebrafish, in spite of the absence of the secondary palate, the palate is also formed in the anterior neurocranium and is composed of the ethmoid plate and trabeculae, which originate from the frontonasal and maxillary prominences, respectively (Figure 8A). Clefting, shortening, or the absence of these structures represent prototypical orofacial abnormalities in zebrafish (Dougherty *et al.*, 2012; Eberhart *et al.*, 2008; Swartz *et al.*, 2011; Wada *et al.*, 2005). Thus, palatogenesis following the PA1 formation in zebrafish and mammals is thought to progress in similar manners.

In addition to the morphological similarity, zebrafish and mammals share similar teratogenic responses (Liu *et al.*, 2020). Furthermore, they utilize the same molecular signaling pathways, including Fgf, Pdgfr, Bmp, Tgfb, Wnt, and Shh pathways, in palatogenesis, and disruption of any of these pathways could result in craniofacial defects in both zebrafish and mammals (Bush and Jiang, 2012; Schilling and Kimmel, 1997). Indeed, in our previous research, cleft palate in zebrafish induced by teratogens was triggered by canonical Wnt pathway inhibition, which is the same etiology of cleft palate as that found in mammals (Narumi *et al.*, 2020). These lines of evidence support the idea that zebrafish share conserved craniofacial morphogenesis and teratogenic responses with mammals. Therefore, zebrafish is considered to be a useful model for evaluating and predicting chemical-induced craniofacial anomalies across vertebrates.

Identification of an AOP has been essential for developmental toxicity evaluation as well as cross-species extrapolations (Ankley *et al.*, 2010; Knapen *et al.*, 2015). Recently, an AOP of developmental vascular toxicity was identified (Saili *et al.*, 2017); however, there is no report of an AOP causing craniofacial anomalies. Developmental toxicity manifests during morphogenesis, and thus teratogenicity should be monitored and evaluated in a chronological manner. Craniofacial anomalies have been investigated mainly by observation of gross external morphology or the pattern of Alcian blue cartilage staining (Brannen *et al.*, 2010, 2013; Liu *et al.*, 2020). Although these strategies have been successful in detecting the readout of teratogenicity, the developmental process for the craniofacial anomalies could not be traced with these methods. To solve this problem, we established a series of *sox10*-reporter transgenic lines in zebrafish, which enables us to monitor for CNCC-based chronological teratogenic response and thereby to elucidate key events along with the causal relationship inducing craniofacial anomalies (Table 1). Several genes including *foxd3* have been identified as markers for CNCCs (Drerup *et al.*, 2009). Among these markers, *sox10* is the most suitable in that it continues to be expressed in craniofacial cartilage after 48 hpf (Carroll *et al.*, 2020), and thus can monitor both CNCC development and craniofacial morphogenesis.

Our *sox10:EGFP* zebrafish line enables us to monitor the gross morphology of CNCC populations during craniofacial morphogenesis (Figs. 1A and 1A′). The *sox10:EGFP-CAAX* line demarks the cell membrane of each CNCC (Supplementary Figs. 1B and 1B′) so that phenotypes can be examined at a cellular level. Finally, the *sox10:Dendra2* line enables tracing of the developmental origin and differentiation fate of CNCCs (Supplementary Figs. 1C and 1C′). These lines can also detect cartilage anomalies at later stages (Figure 2 and Supplementary Figs. 2 and 3). The expression patterns of these fluorescent reporter genes are in agreement with the previously reported *sox10* reporter lines (Askary *et al.*, 2015; Dougherty *et al.*, 2012). Therefore, our lines are suitable for examining the early events of teratogenicity such as migration and apoptosis through live imaging and monitoring the development of CNCCs. Gene expression analysis will be needed to further confirm the usefulness of this system, but it will be a next step to be done in the near future. Taken together, these transgenic lines provide the powerful platform with which to analyze the early events causing craniofacial anomalies and to evaluate AOP-based teratogenicity chronologically.

CNCC development is classified into 3 periods: CNCC migration, PA1 formation and outgrowth of craniofacial placodes. Live-imaging with *sox10:EGFP* lines detected defects in CNCC migration, PA1 formation, and craniofacial morphology as an adverse outcome. When exposed to teratogens, premigratory and migratory CNCCs at 10 ss exhibited decreased proliferation and increased apoptosis, but CNCCs in the PA1during the pharyngeal only showed decreased proliferation at 24 hpf. These results suggest that migratory CNCCs are more vulnerable and/or sensitive to teratogens. Consistent with these results, when zebrafish embryos were treated with the teratogens for 4–24 hpf or 24–96 hpf, craniofacial anomalies occurred more frequently in the 4–24 hpf treatment than in the 24–96 hpf treatment embryos (Supplementary Figure 5). Taken together, we conclude that the migration period is critical for chemical-induced craniofacial anomalies and that the resulting PA1 morphology at the pharyngeal period 24 hpf is a reliable endpoint for predicting chemical-induced craniofacial anomalies.

Finally, we provide the following AOP for chemical-induced craniofacial anomalies (Figure 8B):

1. Molecular response: Disturbance of gene expression during CNCC development
  Our previous study showed that teratogen-exposed embryos showed disturbance of the expression of genes involved in CNCC development (*tfap2a*, *zic2a, pax3*, *snail2*, *sox10*, and *sox9a*) (Liu *et al.*, 2020). Genetic manipulation of some of these genes also induces defects in CNCCs leading to craniofacial anomalies in zebrafish (Knight *et al.*, 2003; McKeown *et al.*, 2005; Minchin and Hughes, 2008; TeSlaa *et al.*, 2013; Yan *et al.*, 2005).
2. Cellular response: Inhibition of CNCC migration, decreased mitotic ability, and increased apoptosis in premigratory and migratory CNCCs
3. Tissue response: Disturbance of PA1 morphogenesis at the pharyngeal stage
4. Adverse outcomes: Craniofacial anomalies such as cleft palate and micrognathia

Taken together, we propose a method for an alternative teratogenicity assay using transgenic zebrafish lines (Figure 8C). Teratogens are administered to *sox10* transgenic lines at 4 hpf. The first endpoint is the PA1 formation at the pharyngeal period. This deformity predicts later craniofacial anomalies. The second endpoint is craniofacial morphology at 96 hpf, which is a readout of the PA1 defect.

In sum, we have revealed the AOP for craniofacial anomalies caused by teratogens using zebrafish transgenic lines and identified cellular key events. Based on highly conserved developmental mechanisms and teratogenic responses between mammals and fish, we assume that our findings can be directly applicable to mammalian embryos. Zebrafish is thus a promising model for evaluating cross-species chemical-induced developmental toxicity.

## Supplementary data

Supplementary data are available at *Toxicological Sciences* online.

## Declaration of conflicting interests

S.L., N.I., M.Y., and J.T. are employed by the company Kao. The author/authors declared no potential conflicts of interest with respect to the research, authorship, and/or publication of this article.

## Supplementary Material

kfad078_Supplementary_Data
