# Supplementary material for: Identification of an adverse outcome pathway (AOP) for chemical-induced craniofacial anomalies using the transgenic zebrafish model
Source: Toxicol Sci. 2023 Aug 2;196(1):38–51. doi: 10.1093/toxsci/kfad078 (PMC10614053; doi:10.1093/toxsci/kfad078)
Supplement: kfad078_Supplementary_Data [file kfad078_supplementary_data.zip › kfad078_Supplementary_Data/toxsci-23-0146-File015.docx]

**Supplemental Video Legends**

Supplemental Video 1

Time-lapse imaging of control embryo from the 10 ss to the 20 ss.

Supplemental Video 2

Time-lapse imaging of valproic acid (VPA)-treated embryo from the 10 ss to the 20 ss. Zebrafish embryos were subjected to treatment with higher dose of valproic acid (30 µM) from the 10 ss to the 20 ss.
