# Supplementary figures and images for: Identification of an adverse outcome pathway (AOP) for chemical-induced craniofacial anomalies using the transgenic zebrafish model
Source: Toxicol Sci. 2023 Aug 2;196(1):38–51. doi: 10.1093/toxsci/kfad078 (PMC10614053; doi:10.1093/toxsci/kfad078)

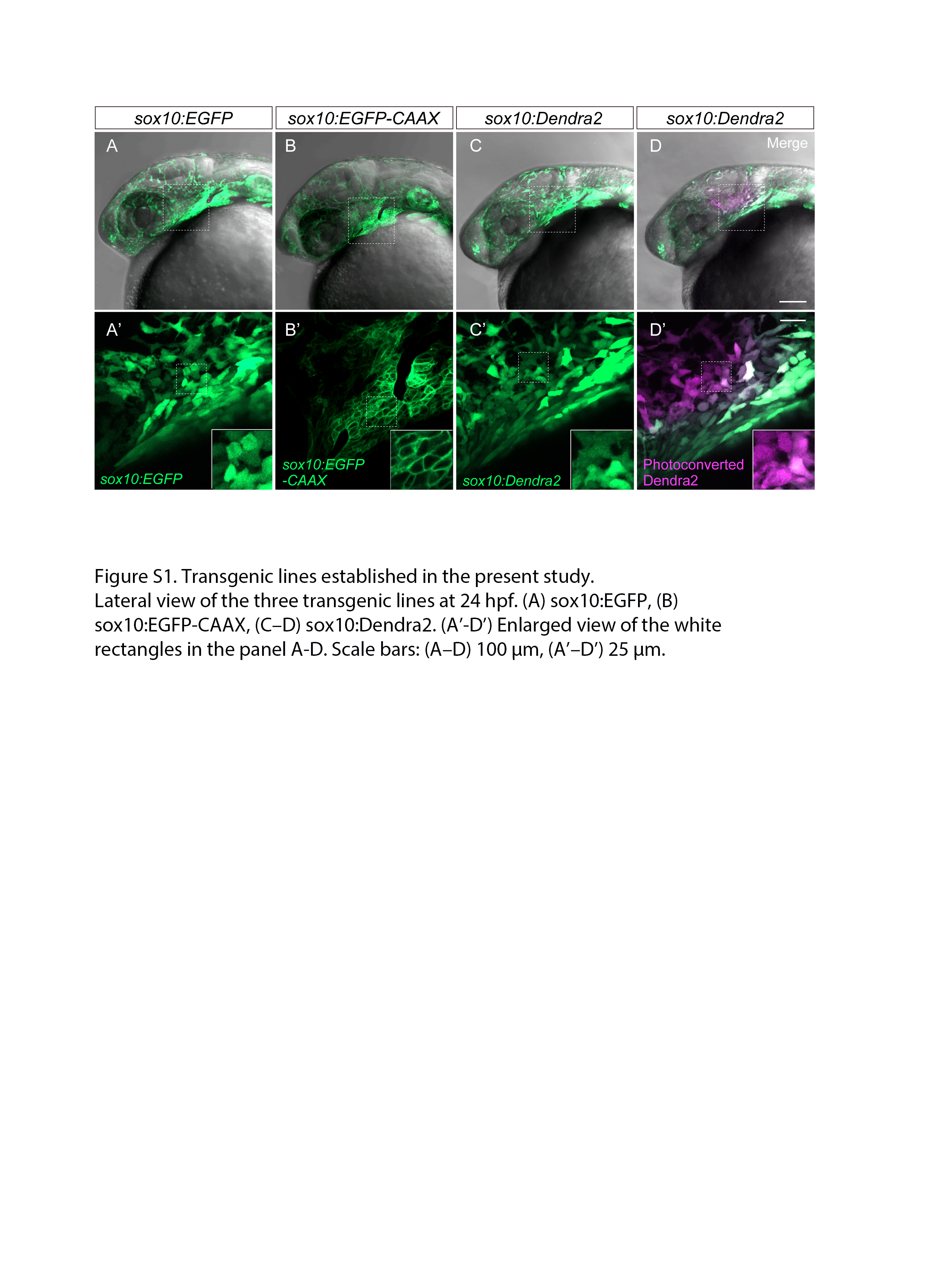

Supplement: kfad078_Supplementary_Data [file kfad078_supplementary_data.zip › kfad078_Supplementary_Data/toxsci-23-0146-File010.tif]

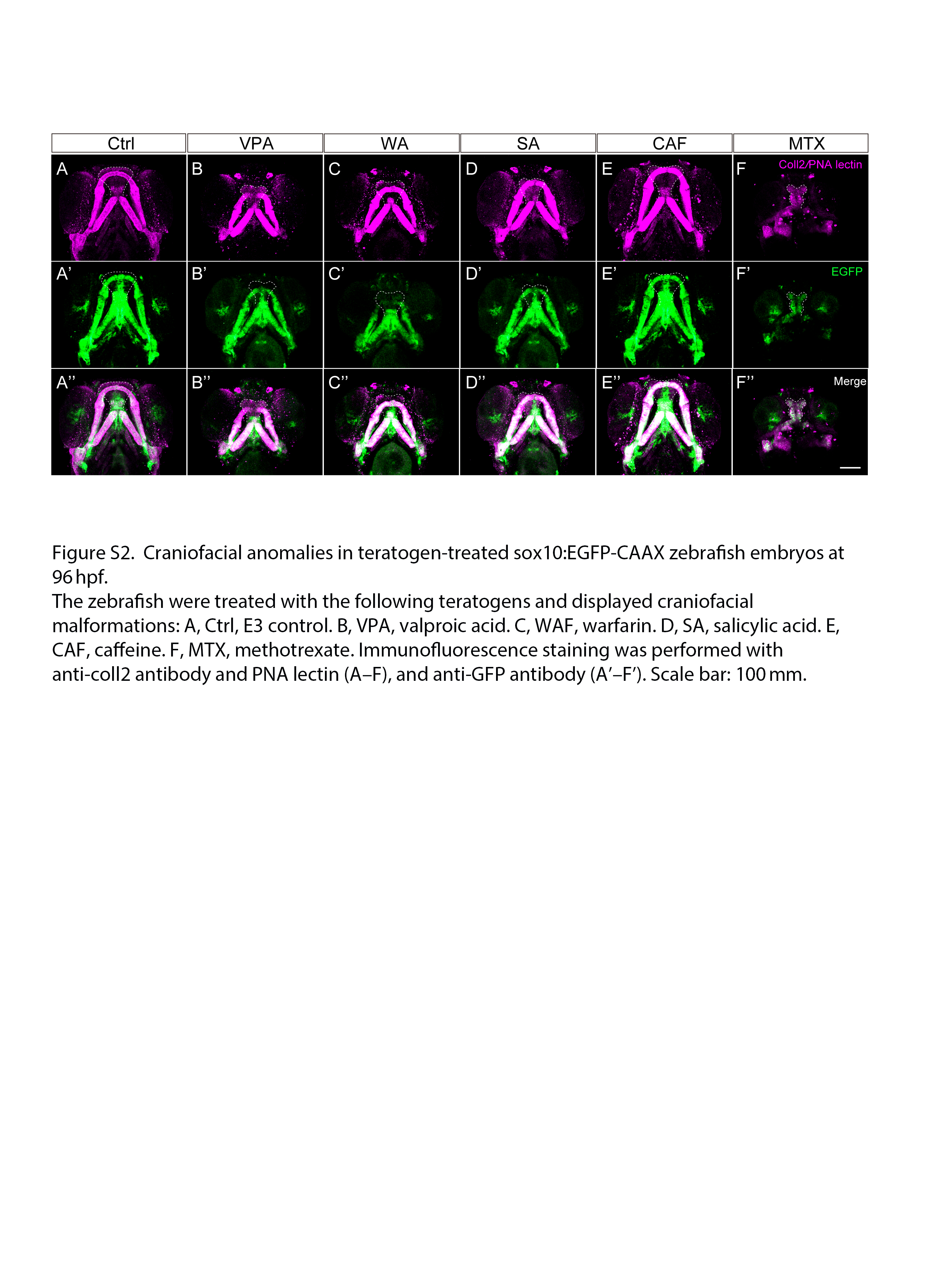

Supplement: kfad078_Supplementary_Data [file kfad078_supplementary_data.zip › kfad078_Supplementary_Data/toxsci-23-0146-File011.tif]

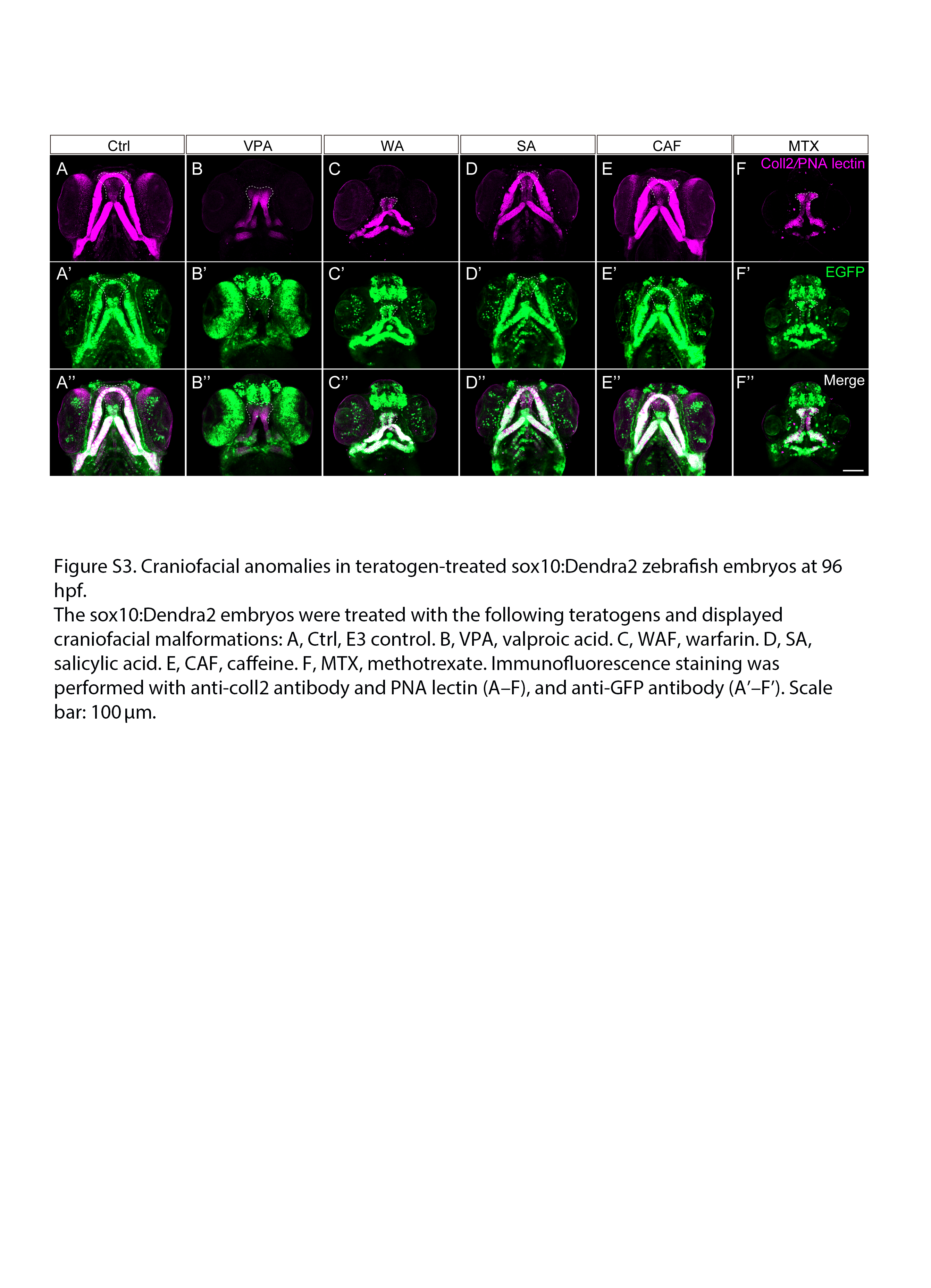

Supplement: kfad078_Supplementary_Data [file kfad078_supplementary_data.zip › kfad078_Supplementary_Data/toxsci-23-0146-File012.tif]

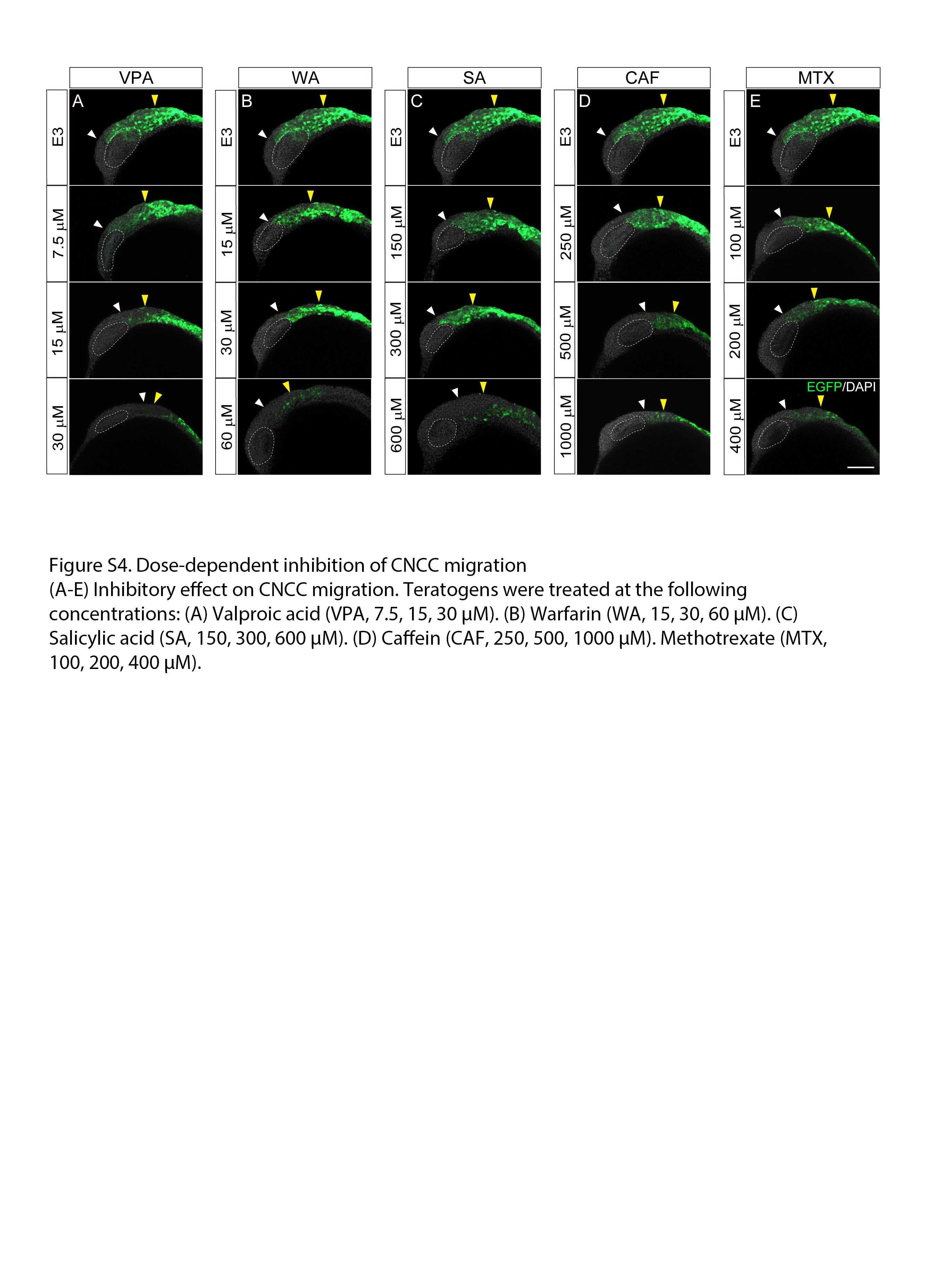

Supplement: kfad078_Supplementary_Data [file kfad078_supplementary_data.zip › kfad078_Supplementary_Data/toxsci-23-0146-File013.tif]

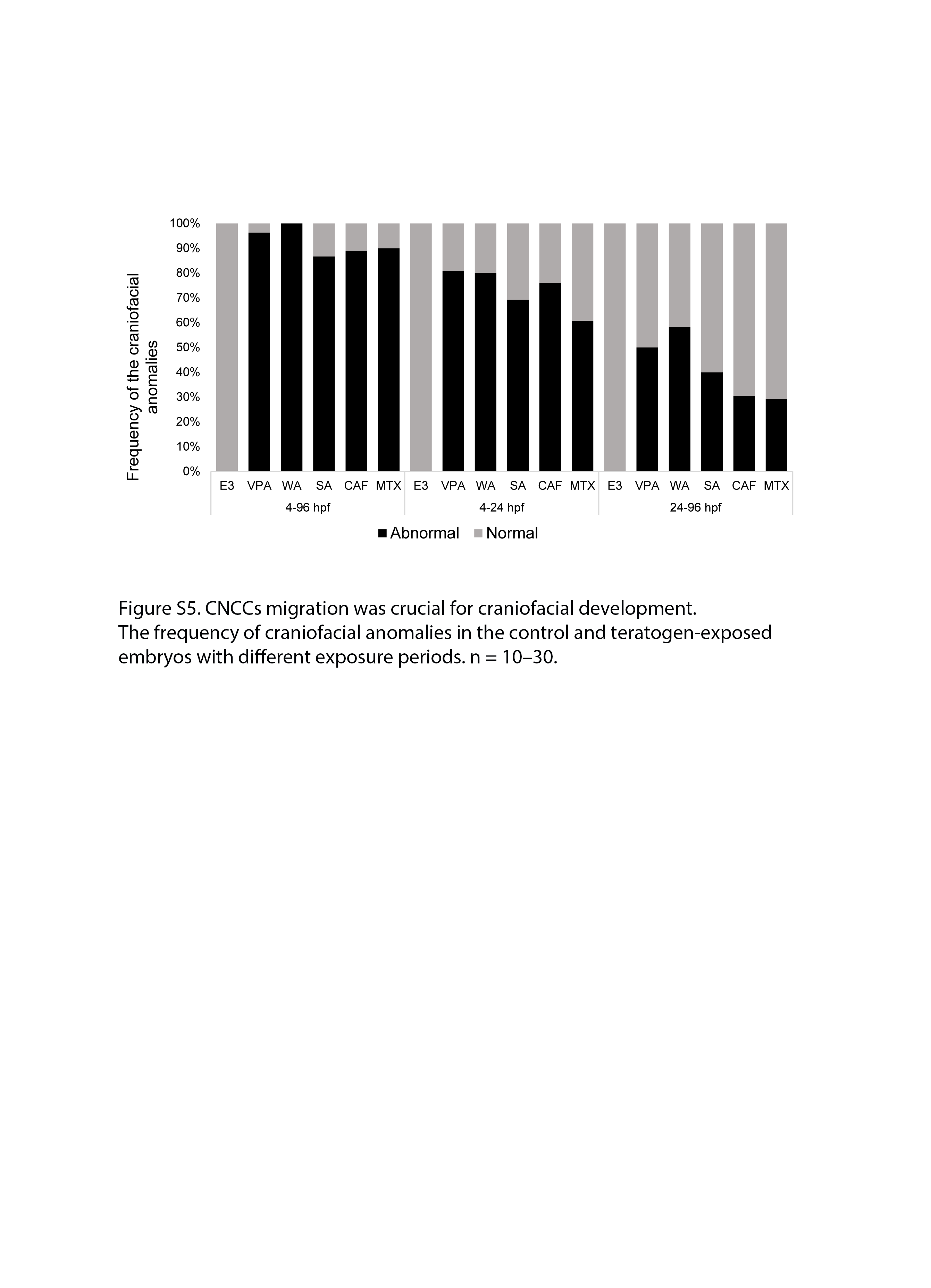

Supplement: kfad078_Supplementary_Data [file kfad078_supplementary_data.zip › kfad078_Supplementary_Data/toxsci-23-0146-File014.tif]
